# Efficiency of Near-Infrared Spectroscopy in Quantifying Lignin in Black Liquor-Impregnated Reforestation Wood

**DOI:** 10.3390/polym17192614

**Published:** 2025-09-27

**Authors:** Luzia Barcelos Deknes, Karen Keli Barbosa Abrantes, Renan Falcioni, Caio Almeida de Oliveira, Glaucio Leboso Alemparte Abrantes dos Santos, Marcos Rafael Nanni, Juarez Benigno Paes, Lúcio Cardozo-Filho

**Affiliations:** 1 Postgraduate Program in Agronomy, State University of Maringá, Av. Colombo, 5790, Maringá 87020-900, PR, Brazil; luziadeknes@gmail.com (L.B.D.); kkelibarbosa@gmail.com (K.K.B.A.); renanfalcioni@gmail.com (R.F.); pg55482@uem.br (C.A.d.O.); mrnanni@uem.br (M.R.N.); 2 Soil and Plant Nutrition, State University of Maringá, Maringá 87020-900, PR, Brazil; glaucioalemparte@gmail.com; 3 Department of Forest and Wood Science, Federal University of Espírito Santo, Jerônimo Monteiro 29550-000, ES, Brazil; jbp2@uol.com.br; 4 Department of Chemical Engineering, State University of Maringa, Av. Colombo, 5790, Maringá 87020-900, PR, Brazil

**Keywords:** cellulose content, high pressure, *Eucalyptus urophylla*, *Pinus taeda*, chemical analysis

## Abstract

Cellulose and lignin are biopolymers with significant potential for chemical synthesis and energy production; however, their heterogeneous composition presents challenges for their use as raw material sources. This study employed near-infrared (NIR) spectroscopy coupled with partial least-squares regression (PLSR) to predict cellulose and lignin content in sapwood and heartwood of *Eucalyptus urophylla* and sapwood of *Pinus taeda*, all impregnated with black liquor under high pressure. Samples were analyzed across three longitudinal sections (top, middle, base), with no significant compositional variation detected. Near-infrared spectral data (1100–2500 nm) and pre-processed using the standard normal variate (SNV) method, yielded high predictive accuracy: *R*^2^ values of 0.98–0.99 for cellulose and 0.94–0.96 for lignin, with root mean square error (*RMSE*) values of 0.2–0.3 and 0.1, respectively. Principal component analysis (PCA) explained 98% of sample variance, revealing clear distinctions between *E. urophylla* sapwood and heartwood. These findings confirm the efficacy of NIR-PLSR as a nondestructive, reliable alternative to conventional chemical analyses, with implications for improved quality control and decision-making in the wood treatment industry.

## 1. Introduction

Sustainable management and expansion of planted forests are crucial strategies for providing renewable resources [1] while safeguarding tropical ecosystems. Eucalyptus is a strategic species for the development of high-yield, fast-growing forests [2]. *Eucalyptus* and *Pinus* spp. are the most utilized species for producing panels, pulp, paper, and sawn timber [3,4]. Despite their good employability, these species have some restrictions on use due to their low natural durability, compared to species with higher density. To increase the resistance and longevity of these species, wood impregnation methods under pressure promise uniform penetration of protective substances into the cellular structure of timber [5].

However, conventional treatments often utilize highly toxic chemicals, such as chromated copper arsenate (CCA), which are restricted in their applications in many regions [5,6]. Several countries, including members of the European Union and the United Kingdom, have enacted laws restricting or prohibiting its use for more than a decade [7,8]. However, CCA continues to account for 80% of the volume of treated wood in Brazil [9]. In response, the use of high pressure for wood impregnation offers an efficient route for the deposition of preservatives minimizing the environmental and health impacts of highly toxic chemicals [10]. Additionally, black liquor—an industrial byproduct from the kraft process in cellulose production—has emerged as an effective alternative due to its robust antioxidant activity and antifungal and insecticidal properties against wood-boring agents [11,12].

Wood comprises two distinct regions: sapwood, characterized by high moisture content and increased susceptibility to biological decay [13,14] and heartwood, formed from dead cells infused with phenolic extractives and tannins, rendering it less vulnerable [14,15,16]. Wood strength is not directly affected by the transition from sapwood cells to heartwood cells [17]. The interplay between the chemical composition and anatomical structure of wood is important, as it directly influences the machinability, mechanical strength, and effectiveness of preservative treatments [18].

Cellulose and lignin are considered biopolymers with significant industrial applications. Lignin can serve as both a renewable biopolymer and a bioactive compound [19]. Cellulose has a range of applications, from the traditional pulp and paper industry to the controlled delivery of drugs [20]. Lignin has gained prominence in cosmetics due to its antioxidant and ultraviolet-blocking properties as a replacement for synthetic additives [21]. Its antimicrobial and antifungal properties enable phytotherapeutic applications [22].

Traditional analytical methods such as the use of acetyl bromide for lignin quantification—have limitations due to the use of toxic reagents, sample destruction, and prolonged analysis times [23]. Furthermore, the chemical characterization of biomass using conventional methods is not applicable on a large scale, due to the high demand for labor and the time required to process these analyses, which is inadequate for interfacing with production systems [24]. However, the emergence of spectroscopy as a nondestructive analytical tool provides exciting alternatives for investigating chemical modifications in treated wood [25]. When paired with chemometric methodologies, spectroscopy can yield predictive insights into the changes in both the chemical and physical structures of wood [26,27]. 

NIR-based prediction models have been developed with applicability in the wood industry. In the pulp and paper sector, studies demonstrate that NIR-based chemometric models can estimate cellulose and lignin content and pulp yield with high accuracy, reducing costs associated with conventional chemical analyses [28]. Recent research includes the use of NIR techniques in predicting various cellular components, such as lignin and cellulose contents in moso bamboo [29], *Eucalyptus dunnii* wood [30], poplar and eucalyptus wood [24]; lignin in *Pinus radiata* [31] and in 5-year-old *Eucalyptus urophylla* × *Eucalyptus grandis* clones [32] and extractives in *Eucalyptus bosistoana* F. Muell [33]. In the field of biotechnology and biorefineries, lignocellulosic content of agricultural biomass is used in the optimization of process conversion into biofuels [33,34].

The synergy of NIR spectroscopy with multivariate analytical methods enables comprehensive. Techniques such as principal component analysis (PCA) facilitate the identification of spectral patterns, aiding in the differentiation of structural and chemical variations between treated and untreated samples [35]. Partial least-squares regression (PLSR) is useful for correlating spectral data with quantitative variables, enabling robust predictive modeling of the chemical and physical-mechanical properties of wood samples [18,26,36]. Compared to traditional reference analyses, near-infrared (NIR) spectroscopy has revolutionized the evaluation of wood and wood products. The technique offers substantial advantages, including reduced operating times, simplified sample preparation, and the ability to analyze numerous samples without hazardous chemicals [18].

The novelty of this research lies in the calibration and validation of robust models that can accurately, precisely, quickly, cost-effectively, and with low environmental impact predict the main cellular components (lignin and cellulose) of reforested timber species and their different regions (sapwood and heartwood) in wood submitted to preservative treatment. Thus, this study aims to validate the effectiveness of non-imaging NIR spectroscopy using a Fieldespec® sensor (ASD Inc., Boulder, CO, USA) to detect key chemical constituents—specifically lignin and cellulose—in reforested species (*Eucalyptus urophylla* and *Pinus taeda*) and their different regions (sapwood and heartwood of *Eucalyptus urophylla*) subjected to high-pressure black liquor impregnation. The results will be compared with traditional chemical analysis methods to assess advances in wood spectroscopy techniques.

## 2. Material and Methods

### 2.1. Origin and Characterization of Black Liquor

The black liquor (BL) used in this study was provided by the Brazilian Pulp and Paper Company, Kablin S.A., which is located in Telêmaco Borba, Brazil. The raw materials utilized included a mixture of wood from hardwood species (*Eucalyptus* spp.) and coniferous species (Pinus spp.). The black liquor used in this study has a high pH of 13.32 and contains 66.65% total soluble solids (Barbosa Abrantes et al., 2023) [11]. Its moisture content is 33.3% and its ash content is 27.67%. The specific woods selected for producing the samples were *Pinus taeda* sapwood, *Eucalyptus urophylla* sapwood, and both the sapwood and heartwood of the latter. The lignin content is 24.9 g/L. The high level of ash may come from inorganic substances in black liquor, such as alkaline salts such as Na_2_S and NaOH, which are commonly used in the Kraft process [37,38].

Cylinders measuring 35 × 17 mm were obtained from *Pinus taeda* sapwood and from both sapwood and heartwood of *Eucalyptus urophylla*. The specimens were shaped according to AWPA (2016) [39] specifications so that size and geometry would remain uniform.

### 2.2. High-Pressure Wood Impregnation

**Figure 1:**
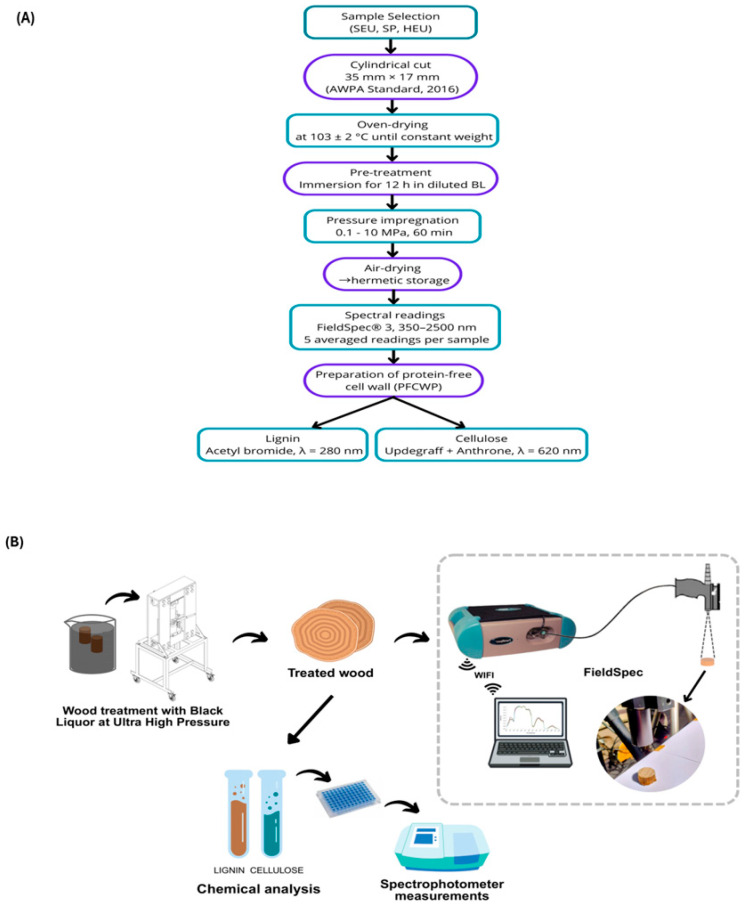
(**A**) Flowchart of the process of selection, preparation, treatment, and chemical characterization of HEU, SEU, and SP samples. (**B**) Schematic illustration of high-pressure wood treatment, hyperspectral readings, and chemical analysis.

The wood samples, cylindrical with dimensions of 35 × 17 mm [40], were prepared oven-dried (Model 400/4ND, Nova Etica, Brazil) at 103 ± 2 °C until constant mass (Figure 1A). A pretreatment step consisted of immersing the specimens in diluted black liquor for 12 h before pressurization in the Ultra Pressurization Unit (UHP) (Figure 1B). The equipment was produced and patented by the Supercritical Technology and Phase Equilibrium Laboratory (LTSEF), in the Department of Chemical Engineering at the State University of Maringa, PR, Brazil. The pretreatment solution prepared by diluting black liquor in water, according to the concentration defined for each treatment. The samples were then transferred to the UHP and treated with 120 mL of black liquor solution at room temperature (approximately 25 °C).

The impregnation process was carried out at pressures defined by the experimental design: 0.10, 2.5, 5.0, 7.5, and 10 MPa. After the samples were placed inside the UHP vessel and sealed, the stabilization phase was initiated, and the treatment time of 60 min was recorded from that point. Following impregnation, the samples were air-dried under ambient conditions until they reached constant weight and subsequently stored in airtight containers. After 48 months of storage, chemical analyses were conducted to evaluate preservative retention. The specimens were treated with black liquor and subjected to six different pressure conditions: untreated control, 0.1, 2.5, 5.0, 7.5, and 10 MPa. Five samples were selected for each pressure level and species.

### 2.3. Hyperspectral Sensors

We conducted hyperspectral analyses on wood via a FieldSpec®3 UV-VIS-NIR (350–2500 nm) spectroradiometer portable (ASD Inc., Boulder, CO, USA) and an ASD contact PlantProbe (ASD Inc., Boulder, CO, USA). Both devices were calibrated and set up before standard white reference plates were used. We applied a light beam of approximately 2000 μmol/(m^2^ s) to take reflectance readings from 350 to 2500 nm [36]. We took five readings from the cross-section for each wood sample and averaged them to form one experimental unit. We collected absorbance data from the near-infrared range of 1100 to 2500 nm. We analyzed the correlations between the spectral data and the chemical tests of lignin and cellulose for each sample, considering the species, pressure levels, and sections of the wood, versus the untreated control (Figure 1).

### 2.4. Chemical Analysis

#### 2.4.1. Protein-Free Cell Wall Preparation (PFCWP)

We prepared 270 wood samples by sanding them and sifting them through a 35-mesh screen (425 µm). Portions of 0.15 g were suspended in 50 mM potassium phosphate buffer (1.85 mL, pH 7.0) and centrifuged at 1,5000 rpm for 5 min in a Mikro 200R centrifuge (Andreas Hettich GmbH & Co., KG, Tuttlingen, Germany). The pellets were sequentially washed: four with buffer, four times with 1% (v/v) Triton X-100, three with 1 M NaCl in buffer, three with distilled water, and twice with acetone. Samples were dried at 40 °C for 24 h, resulting in a protein-free cell wall fraction (PFCW) [41].

#### 2.4.2. Determination of Lignin in PFCW

To determine the lignin content, we treated 0.10 g of the PFCW samples with 0.5 mL of acetyl bromide (25%, v/v in glacial acetic acid) and incubated them at 70 °C for 30 min in a dry bath. After cooling on ice, we added NaOH (2 M) and hydroxylamine-HCl (5 M) to glacial acetic acid, resulting in a total volume of 2 mL. We then transferred the resulting mixture to 96-well ELISA microplates and measured the absorbance at 280 nm using a FlexStation 3 plate spectrophotometer (Molecular Devices LLC., San Jose, CA, USA). We created a standard curve using alkaline, standard lignin (Aldrich 37.0967) to calculate the molar absorptivity. The results were reported as milligrams of lignin per gram of cell wall [42]. The curve included 12 concentration points (0–400 µg/mL; 0, 20, 40, 60, 80, 100, 120, 140, 160, 180, 200, 400 µg/mL), analyzed in duplicate. The calibration model achieved *R*^2^ = 0.87. The results were reported as milligrams of lignin per gram of cell wall.

#### 2.4.3. Determination of Cellulose in PFCW

We estimated the crystalline cellulose content via the Updegraff method [23]. We collected samples (0.10 g) and washed them with 70% ethanol, 100% methanol, chloroform/methanol (1:1), and 100% acetone. We then heated the samples to 70 °C and spun them at 15,000 rpm for 10 min. After drying in an oven at 40 °C for 24 h, the samples were treated with the Updegraff reagent (a mixture of H_2_O, 80% acetic acid, and nitric acid) at 70 °C for 30 min. We followed this with centrifugation and a wash with acetone. The final samples were dried in an oven at 37 °C for 12 h. We added 67% sulfuric acid to the samples for cellulose hydrolysis and mixed them for 1 h. Released glucose was quantified using 2% Anthrone reagent, with absorbance read at 620 nm in MDVersaMax ELISA microplate reader (Molecular Devices LLC., San Jose, CA, USA). Standard curves were prepared from glucose stock solutions with 12 points (0–400 µg/mL) in duplicate, yielding *R*^2^ = 0.96. Results were expressed as glucose equivalents (µmol/g cell wall). Based on this standard curve, we expressed the results as glucose concentration equivalent (µmol glucose per gram).

### 2.5. Univariate and Multivariate Statistical Analysis

The experimental design included three types of wood specimens: heartwood of *E. urophylla* (HEU), sapwood of *E. urophylla* (SEU), and sapwood of *P. taeda* (SP), subjected to six different pressure conditions (untreated control plus five treatment levels). Each specimen was sectioned into three parts (top, middle, and base) to allow longitudinal analysis. Five replicates were prepared for each species × pressure combination. For each sample, two spectral readings were taken for lignin and cellulose. This scheme resulted in 90 analytical units per section, totaling 540 data points for the entire experiment (Table A1).

Spectral data were preprocessed by the Standard Normal Variate (SNV) method to reduce scattering and thickness effects [43]. Multivariate analyses were performed using The Unscrambler X software, version 10.4 (Camo Software, Oslo, Norway). Principal Component Analysis (PCA) was applied to explore the data structure, while Partial Least-Squares Regression (PLSR) was used to model the relationships between the spectra and the chemical results. Models were built using the NIPALS algorithm and validated using full cross-validation, using 70% of the data for calibration and 30% for independent prediction. Outliers were detected with Hotelling’s *T*^2^ test at the 5% level. The graphs showed distinct clusters in green, blue, and red, with a significant level of *p* < 0.90. We split the dataset into two groups for the Partial Least-Squares Regression (PLSR) model: 70% for calibrating and cross-validating, and 30% for independent prediction.

The PLSR model analyzed lignin and cellulose chemical parameters for SEU, SP, and HEU. To assess performance, we checked coefficients of determination (*R*^2^) and root mean square error (*RMSE*) during calibration, cross-validation, and prediction. We also calculated the performance-to-deviation ratio (*RPD*) from *R*^2^ values for calibration (*R*^2^*C*), cross-validation (*R^2^CV*), and prediction (*R*^2^*P*), as described by Nanni et al., 2018 [44].

## 3. Results and Discussion

### 3.1. Descriptive Biochemical Parameters

The descriptive analysis of lignin and cellulose contents revealed slight variations among the different wood sections (top, middle, and base), considering the distinct tree species (SEU, SP, and HEU) and the applied operational pressures. An analysis of the 540 samples revealed that the lignin and cellulose contents were influenced by the processing conditions and the wood species evaluated. Table 1 shows the mean values and coefficients of variation for lignin and cellulose concentrations in each wood section, regardless of species and operational pressure.

The chemical analyses of the three timber species (SEU, SP, and HEU), operational pressures (0, 0.1, 2.5, 5.0, 7.5, and 10 MPa), and wood sections (top, middle, and base) revealed substantial variability (Table 1). Cellulose and lignin contents were highly consistent, with coefficients of variation (*CV*) ranging from 58.5% (base) to 61.2% (top) for cellulose, and from 28.1% (top) to 30.3% (middle) for lignin. The base section showed the lowest variability in cellulose content (*CV*: 58.5%), while the top section showed the lowest variability in lignin content (*CV*: 30.3%). This pattern was expected, given the pronounced variability between species and operating impregnation pressures. SEU and SP, being sapwood species, allow for easier penetration of impregnating agents than HEU, a refractory species with low permeability under conventional impregnation methods.

Lignin concentrations varied slightly among the sections, with mean values of 30.1% (*CV*: 28.1%) in the top section, 29.6% (*CV*: 30.3%) in the middle section, and 27.7% (*CV*: 29.1%) in the base section. Similarly, cellulose presented stable values among the sections, with values of 2.4% (*CV*: 61.2%), 2.4% (*CV*: 59.5%), and 2.3% (*CV*: 58.5%) for the top, middle, and base sections, respectively. The stable CV% values among the sections, predominant in both wood components (lignin and cellulose), indicate a more homogeneous distribution or a consistent biosynthetic mechanism among the timber species examined [41]. Another important factor is related to sample size, which, being small, did not present significant variations among the sections.

Cellulose is a natural biopolymer found in the cell walls of plants. It has various applications in textiles, packaging, food preservation, wastewater treatment, medicines, and cosmetics [45]. Lignin is the most abundant natural aromatic polymer, present in the vegetable cell wall [46]. Its properties—good thermal stability, high biodegradability, and high carbon content—make it promising for chemical synthesis and energy generation [47]. Lignocellulosic plant biomass varies by cell type and plant taxon [31]. It provides a range of chemical compounds (acids, alcohols, aldehydes, phenolic compounds, polymers, proteins) for adhesives, solvents, food additives, flavors, and fragrances [48]. However, its high variability creates restrictions for use and standardization due to its heterogeneous nature and polymer composition [19]. Fast, accurate, and low-cost methods to quantify natural biopolymers are essential for developing sustainable products and applications. Real-time monitoring of these components optimizes wood processing for industrial purposes.

### 3.2. Principal Component Analysis of Cross-Sections of Different Timber Species

**Figure 2:**
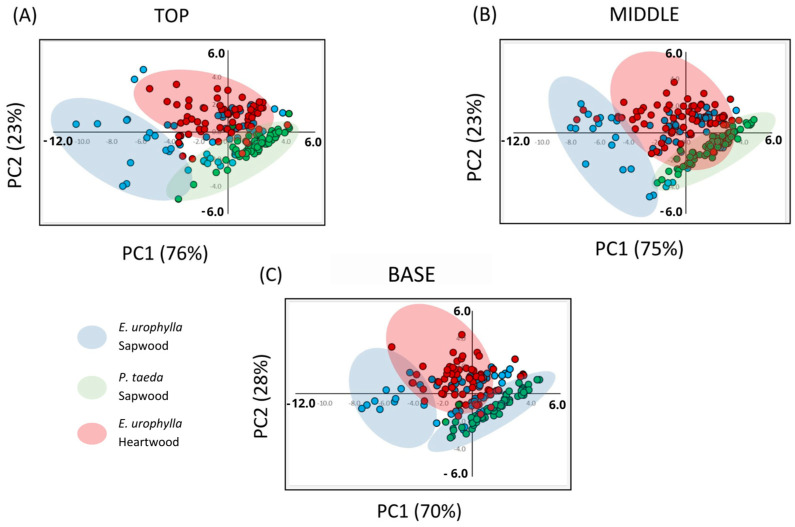
Principal component analysis (PCA) illustrating the variability of hyperspectral data for *E. urophylla*, *P. taeda* sapwood, and *E. urophylla* heartwood at different operating pressures (0, 0.1, 2.5, 5.0, 7.5, and 10 MPa) categorized by section: (**A**) Top; (**B**) Middle; (**C**) Base.

We used principal component analysis (PCA) to assess how cellulose and lignin contribute to the separation of wood sections (top, middle, and base) and timber species (SEU, SP, and HEU), regardless of the operational impregnation pressures (atmospheric, 1.0, 2.5, 5.0, 7.5, and 10 MPa). We visually represented the cumulative variability of the components via clusters (Figure 2), which confirmed that the first two principal components (PC1 and PC2) primarily captured the spectral differences in chemical composition (lignin and cellulose) across different timber species and wood regions (sapwood and heartwood).

The clustering pattern shown in Figure 1 differentiates the timber species (SEU, SP, and HEU) based on their chemical composition. The blue markers represent the sapwood of *Eucalyptus urophylla* (SEU), the red markers represent the heartwood of *E. urophylla* (HEU), and the green markers correspond to the sapwood of *Pinus taeda* (SP). For the top section, the first two principal components (PC1 and PC2) together accounted for 99% of the total data variability, with PC1 accounting for 76% and PC2 accounting for 23%. In the middle section, PC1 and PC2 jointly accounted for 98% of the variability, with PC1 contributing 75% and PC2 contributing 23%. For the basal section, PC1 and PC2 also explained 98% of the variability, with PC1 accounting for 70% and PC2 accounting for 28%.

In Figure 2A–C, distinct cluster formation is evident, particularly for the different woody regions (sapwood and heartwood) of the genus *Eucalyptus*. The most pronounced differentiation corresponds to the HEU region (red), in contrast to the SEU regions (blue) in all sections, with reduced dispersion from the central section. The cluster representing SP (green) remains consistently separated and stable across sections, while the SEU cluster (blue) presents less aggregation and greater variability across sections. The two distinct regions of the wood are called heartwood and sapwood [49]. Sapwood contains a greater amount of polysaccharide compounds and is virtually free of tyloses and phenolic extractives [49]. Physical factors, such as the higher proportion of voids in sapwood than in heartwood, likely explain the greater viability of sapwood [50]. The heartwood, due to its anatomical characteristics, composed of resinous and phenolic extracts that fill its cavity-bound cavities, is responsible for obstructing the flow of liquids [51]. Furthermore, eucalyptus vessels contain numerous tyloses that block the longitudinal movement of liquids [52].

The heartwood region is considered problematic in some industrial processes, such as the delignification process in the pulp and paper industry. Woods with heartwood can result in higher chemical consumption and deposition of extractives during the process [53,54]. Differentiation between heartwood and sapwood lignin’s is not adequately achieved by traditional methods [53]. Confocal Raman microscopy (CRM) was able to characterize lignin in the heartwood, sapwood, and bark of multipurpose clones of *E. urophylla* x *E. grandhis* [53].

Principal component analysis (PCA) provides an efficient approach for analyzing hyperspectral data derived from spectral curves [41]. The PCA results accounted for 99%, 98%, and 98% of the variability across species (*Eucalyptus urophylla* and *Pinus taeda*) and wood regions (sapwood and heartwood). PCA differentiated distinct biochemical compositions in terms of lignin and cellulose contents between these regions. This analysis highlights the inherent spectral variability and highlights the substantial contribution of the initial principal components to the total variance in the dataset. In this work, we demonstrated that NIR techniques produced robust predictive models for distinguishing the chemical composition relative to lignin and cellulose content, for distinct regions of the wood (sapwood and heartwood).

### 3.3. Calibration, Cross-Validation and Prediction of the Cellular Components of Timber Species Sections

We applied Partial Least-Squares Regression (PLSR) for data calibration and cross-validation, correlating hyperspectral reflectance with lignin and cellulose composition across different wood species and regions (Table 2). The models for predicting lignin content produced coefficient of determination (*R*^2^) of 0.96 (three sections) in the calibration phase; after cross-validation yielding adjusted values of 0.95, 0.94, and 0.94, respectively. The closer the *R*^2^ value is to 1, the better the model fit, with one indicating that the model explains 100% of the data variability. The *R*^2^ values for the lignin predictive models exceeded 0.90, indicating a strong model fit. Despite a slight reduction in *R*^2^ values after cross-validation (approximately 0.02 points from the calibration value of 0.96), the results suggest that the models are robust and broadly applicable.

For the cellulose predictive models, the coefficient of determination (*R*^2^) reached 0.99 (three sections) and adjusted to 0.98, 0.99, and 0.98, respectively, during cross-validation. The minimal variation between the calibration and validation values reinforces the robustness of the model, with *R*^2^ values exceeding 0.95 indicating high predictive accuracy for cellulose [55]. According to the criteria proposed by Minasny et al. 2013 [56], predictions with *R*^2^ values above 0.75 are considered excellent, those between 0.75 and 0.50 are acceptable, and those below 0.50 are poor. The models achieved high predictive accuracy for cellulose (*R*^2^ = 0.99) and lignin (*R*^2^ = 0.96).

The root mean square error (*RMSE*) quantifies the difference between the predicted and observed values. For the lignin predictive models, the *RMSE* value during calibration was 0.1 across all the sections. The cross-validation (*RMSE-CV*) values were 0.2, 0.2, and 0.1 for the top, middle, and base sections, respectively. The cellulose predictive models exhibited calibration *RMSE* values of 0.3, 0.2, and 0.2 for all sections, with cross-validation yielding identical results. A comparison of the *RMSE* values from the calibration and validation revealed minimal differences for both components, indicating robust models with consistent predictive capacity.

**Figure 3:**
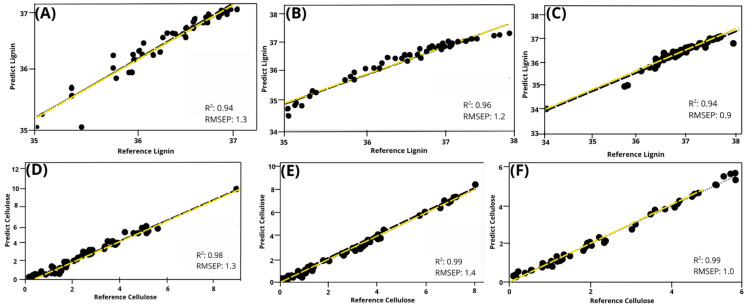
Scatter plots of reference versus predicted data via partial least-squares regression (PLSR) for chemical parameters in wood. Model performance metrics, including the coefficient of determination (*R*^2^) and root mean square error of prediction (*RMSEP*), are provided. The models were trained using 70% of the data for calibration and validated using the remaining 30%. Bias values were consistently below 0.01 and are not shown. (**A**) Lignin top section; (**B**) lignin middle section; (**C**) lignin base section; (**D**) cellulose top section; (**E**) cellulose middle section; (**F**) cellulose base section.

The PLSR models used to estimate cellulose in different sections of wood had up to seven factors, and up to four factors for the lignin predictive model. The *RPD* values well above the acceptable level (>2.0). The slopes were close to ideal, and most models had no bias, indicating strong performance in predicting cellulose and lignin. The statistical parameters of the models’ goodness-of-fit (maximum partial least-squares factor—*PLS*; correlation coefficient—*r*; slope; offset; standard error of prediction—*SEP*; *RPD*; standard deviation, and the linear equation relating the prediction to the calibration model—*R*^2^*P*) shows in Table 3. The accuracies of these PLSR models are shown in scatter plots on Figure 3.

The model adjustment parameters obtained in this work to predict the chemical composition of *Pinus* and *Eucalyptus* species are similar or superior (*R*^2^, *RMSE*, and *RPD*) to other studies using NIR to evaluate the chemical composition of wood and derivatives. A model to evaluate the lignin content in *Eucalyptus urophylla* and *Eucalyptus globulus* obtained *R*^2^ values of 0.95 to 0.86 [57]. The cellulose content of *Eucalyptus nitens*, aimed at increasing genetic gains in Kraft pulp, reported *R*^2^ values ranging from 0.61 to 0.99 [58]. Predictions for lignin and cellulose contents in *Eucalyptus dunnii* indicated *R*^2^ values of 0.87 and 0.83, respectively [30]. PLSR models for juvenile sapwood from *Eucalyptus urophylla* clones presented *R*^2^ values of 0.70 to 0.94, with *RMSE* of 0.55% and *RPD* of 3.3 [59].

Fahey et al. (2018) [31] used NIR to predict the lignin and monosaccharide content of *Pinus radiata*, obtaining *R*^2^ values ranging from 0.97 to 1.0 for different wood particle sizes. The chemical composition and extractive content of 5-year-old *Eucalyptus urophylla* × *Eucalyptus grandis* clones were validated using NIR models, which obtained *R*^2^ values of 0.98 for the standard deviation correction (SNV) treatment [32]. The consistent and robust performance across multiple parameters reinforces the potential of non-imaging hyperspectral analysis combined with PLSR for chemical evaluation, particularly in predicting lignin and cellulose contents across different wood sections and timber species.

In recent years, its application has been successfully demonstrated in various forestry contexts. Among these, the classification of Amazonian wood residues for energy generation has been performed [60], as well as the rapid estimation of mechanical properties—such as the modulus of elasticity and modulus of rupture—of different pine species, with performance surpassing that of traditional methods [61]. Additionally, it is possible to identify and classify timber species [62], along with the estimation of physical and chemical properties of wood [59,63]. Quality control processes have also been supported by the real-time monitoring of wood and paper properties [60,64]. Furthermore, the estimation of quality parameters of charcoal for industrial applications has been conducted using NIR spectroscopy [65].

Spectral reflectance values for the species (HEU, SP, and SEU), observed in the UV-VIS-NIR-SWIR bands, revealed inherent differences in cellulose and lignin contents among species. A significant advantage of NIR-based methods is their ability to measure multiple properties simultaneously [66]. Researchers have shown that greater variability in a property increases the likelihood of obtaining a higher coefficient of determination in calibration models [67]. Polysaccharides and lignin exhibit similar spectral bands, necessitating the application of advanced hyperspectral modeling and multivariate analysis techniques [68]. Although NIR spectroscopy does not directly quantify lignin or cellulose, it allows the detection of variations in wood functional groups. This capability allows researchers to robustly estimate the concentrations of these components through combined multivariate analysis [55].

### 3.4. Spectral Curves (NIR) for Each Wood Section and Wood Species

Chemical characterization of wood by near-infrared (NIR) spectroscopy allows rapid, non-destructive, and highly reproducible analyses [18]. Previous studies have demonstrated that the technique can be used to identify spectral peaks characteristic of lignin and cellulose, aiding in the assessment of their concentrations in treated wood [69]. In this study, the spectral region from 1100 to 2500 nm was defined for the prediction of spectroscopic models and multivariate analysis, based on peaks already established in the literature for lignin and cellulose.

We used the hyperspectral reflectance of the control (untreated wood samples) from each species (HEU, SP, and SEU) as a reference to evaluate species behavior on the basis of their spectral curves and to quantify the functional groups of lignin and cellulose at their characteristic peaks: 1672 nm [29,31] and 2200 nm [18] for lignin, and 1597 and 2343 nm [18,31] for cellulose. The region between 2343 and 2361 nm was associated with the stretching of C-H bonds in cellulose, and 1597 nm is associated with O-H bonds in cellulose [18].

#### 3.4.1. Top

**Figure 4:**
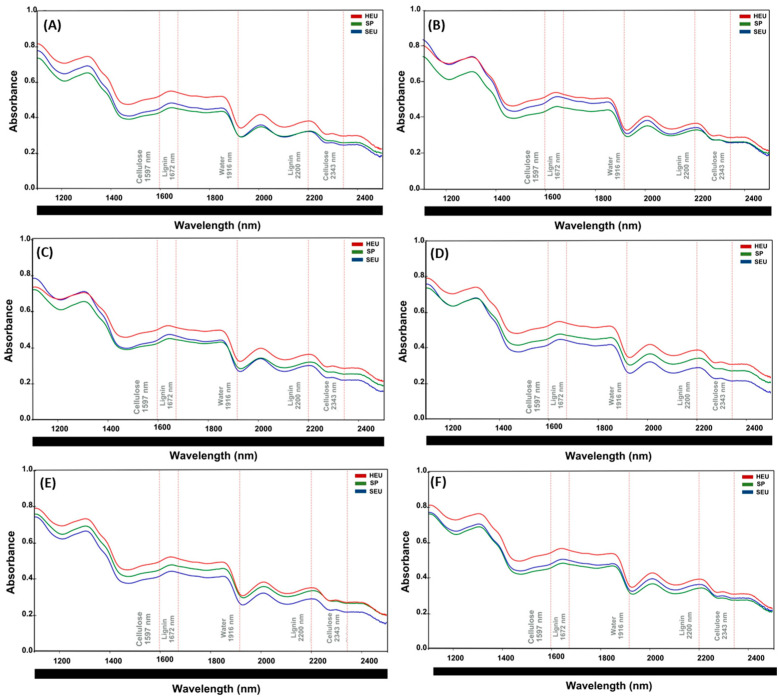
The top section presents reflectance spectra for each species (SEU, SP, and HEU) at different operating pressures: (**A**): Control; (**B**): Atmospheric pressure; (**C**): 2.5 MPa; (**D**): 5.0 MPa; (**E**): 7.5 MPa; (**F**): 10 MPa. HEU is represented in red, SP in green and SEU in blue (*n* = 180).

The spectral curves (Figure 4) illustrate the differences in the hyperspectral reflectance spectra for each species (SEU, SP, and HEU) in the top section, as well as for the various operating pressures (0.1, 2.5, 5.0, 7.5, and 10 MPa).

In terms of the wavelength peaks associated with lignin and cellulose, HEU (as shown in Figure 4A–F) presented stable, higher hyperspectral reflectance values across all treatment pressures than did to SEU and SP. The heartwood of *Fagus sylvatica* L. presented greater lignin and hemicellulose contents than did the sapwood of the same species [70]. SEU generally had higher hyperspectral reflectance values at the wave-length peaks corresponding to lignin than did SP. However, there were exceptions for cellulose at pressures of 2.5, 5.0, and 7.5 MPa (1597 nm) and at 10 MPa (2343 nm), where the hyperspectral reflectance values for the cellulose peaks were greater for SP than for SEU.

#### 3.4.2. Median

**Figure 5:**
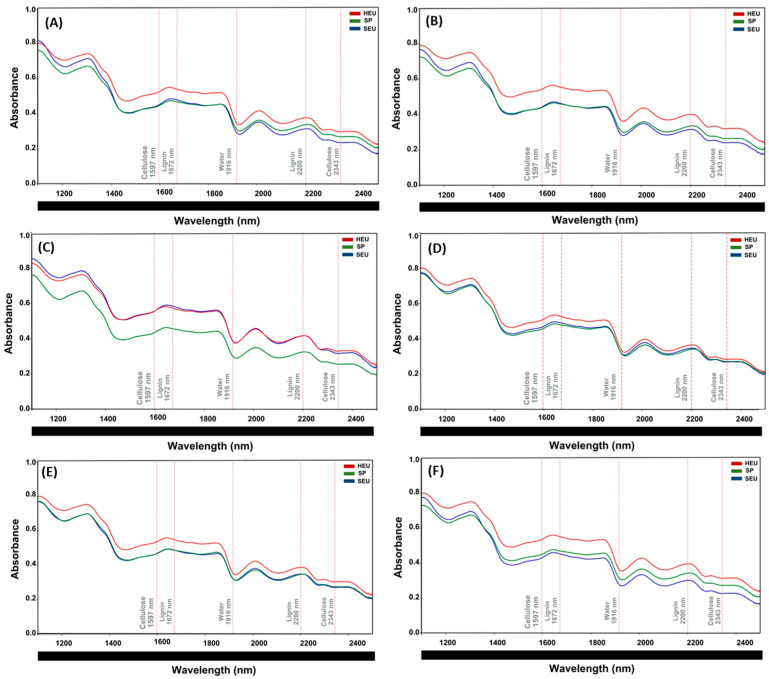
The middle section presents reflectance spectra for each species (SEU, SP, and HEU) at different operating pressures: (**A**): Control; (**B**): 0.1 MPa; (**C**): 2.5 MPa; (**D**): 5.0 MPa; (**E**): 7.5 MPa; (**F**): 10 MPa. HEU is represented in red, SP in green and SEU in blue (*n* = 180).

For the median section of the different wood species and operating pressures (Figure 5A–C), the samples presented differences in their reflectance spectra compared to those of the control. In terms of the wavelength peaks corresponding to lignin and cellulose, HEU (Figure 5A–F) remained stable at all operating pressures, compared with SEU and SP, for the cellulose peaks, and an increase in the lignin peaks (1672 and 2200 nm) was observed. SEU exhibited a reduction in the hyperspectral reflectance values at all pressures, although these values were predominantly greater than those for SP. The exceptions, similar to the apical section, with an increase in hyperspectral reflectance values of SP compared with those of SEU at pressures of 7.5 and 10 MPa (2343 nm).

#### 3.4.3. Base

**Figure 6:**
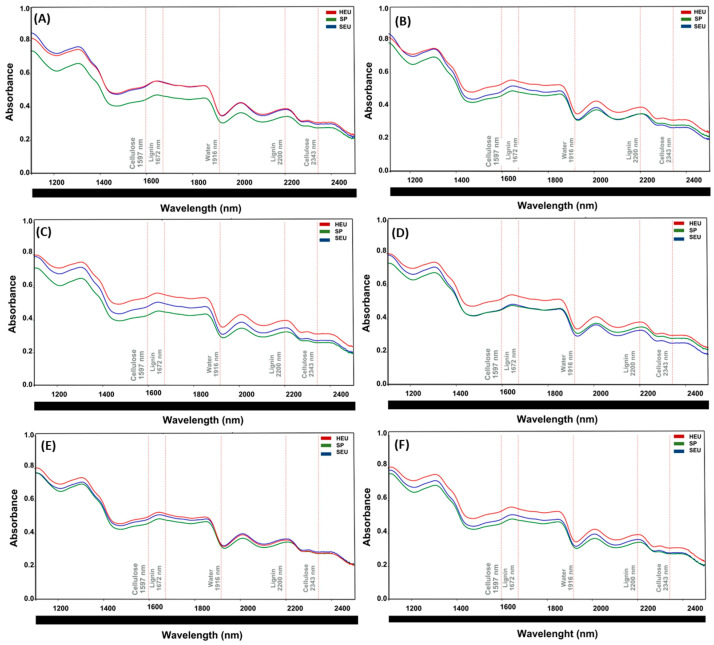
The base section presents reflectance spectra for each species (SEU, SP, and HEU) at different operating pressures: (**A**): Control; (**B**): Atmospheric pressure; (**C**): 2.5 MPa; (**D**): 5.0 MPa; (**E**): 7.5 MPa; (**F**): 10 MPa. HEU is represented in red, SP in green and SEU in blue (*n* = 180).

For the base sections of the different wood species and operating pressures (Figure 6A–C), the reflectance spectra of the samples differed from those of the control samples. There was an increase in the reflectance at the lignin and cellulose wavelength peaks for HEU (Figure 6A–F) at all the treatment pressures, compared with those of SEU and SP, and in the stability of the representative cellulose peaks. We identify an exception at a pressure of 7.5 MPa, where the reflectance at the lignin peak was greater for SEU than for the other species (HEU and SP) at the peak corresponding to the lignin wavelength (2200 nm).

The three species exhibited distinct behaviors under pressure. The HEU exhibited greater resistance to pressure variations, whereas the SEU was more susceptible. In HEU, lignin (1672 nm) and cellulose (1597 nm) had the highest reflectance peaks compared with those of SEU and SP. SEU’s reflectance peaks varied significantly under different pressures, depending on the wood section. SP demonstrated greater stability and predictive behavior, making it suitable for industrial applications. Comparative analysis of wood sections reveals distinct patterns in hyperspectral reflectance. The top section displays high variability, with significant differences between species and increased sensitivity to pressure. The middle section is more stable, showing less variation among species. In the base section, the absorption levels are low and consistent, with minimal divergence. Visual analysis revealed a slight shift in the peaks for lignin (1672 and 2200 nm) and cellulose (1597 and 2343 nm) across all the sections, although their positions remained largely unchanged. For cellulose, the middle section shows similar reflectance values for HEU, SEU, and SP.

The spectral curves revelated that HEU presented the highest reflectance values across all sections (top, middle, and base). Abe et al. 2022 [71], evaluating the reflectance in 21 species of conifers in the range of 200–1200 nm, observed higher reflectance values for the heartwood, compared to the sapwood. Heartwood, which is composed of inactive and aged cells, contains cellular elements filled with extractives that naturally obstruct light transmission, thereby reducing absorbance and increasing reflectance. Density of wood and secondary metabolites in heartwood can reduce the transmittance of light [71].

In general, SP presented lower reflectance values, with minor exceptions under specific pressure conditions (2.5 and 5.0 MPa). Longitudinal tracheids constitute approximately 90% of the sapwood volume [72]. Although the wood cell wall scatters light, an increased median pore area can cause multiple reflections, reducing transmitted light intensity [73], which may explain the lower reflectance observed in sapwood.

Compared with those of heartwood, the spectra of sapwood samples presented greater showed higher absorbance and, consequently, lower reflectance. The higher moisture content in sapwood causes it to absorb more NIR radiation, which reduces its reflectance [74]. Open tracheid’s on the transverse surface and the presence of rays on the tangential surface allow light to penetrate deeper into the wood, leading to lower reflectance [75]. The higher moisture content found in sapwood increases near-infrared absorbance [30]. Additionally, researchers have successfully combined NIR spectroscopy with partial least-squares regression (PLSR) to correlate spectral data with quantitative variables. This methodology enables robust predictive modeling of the chemical and physical-mechanical properties of wood samples [18,26,36].

The results obtained in this study for the three species and different operating pressures demonstrate that applying the standard normal variate (SNV) method to normalize spectra, in conjunction with selecting wavelengths in the 1100–2500 nm range, enhances the predictive performance of models for the evaluated chemical parameters. Hein et al. (2010) [76] reported similar findings when they used the first and second derivatives of spectra to calibrate models for estimating the basic density and modulus of elasticity in parallel compression tests of Eucalyptus wood fibers. Notably, the absorption bands related to the chemical components of wood remain consistent regardless of measurement conditions, suggesting that intrinsic wood information can aid in species classification via NIR spectroscopy in both stationary and dynamic environments [75].

## 4. Conclusions

The models accurately predicted subtle variations in lignocellulosic components across wood species (Eucalyptus and Pine) and regions (sapwood and heartwood), as evidenced by high coefficients of determination (*R*^2^ = 0.94–0.99) and low *RMSEP* values (0.1–0.3) for lignin and cellulose across sections. The consistent performance highlights the effectiveness of imageless hyperspectral analysis combined with partial least-squares regression (PLSR) for non-destructive chemical assessment. Principal component analysis (PCA) further revealed distinct spectral patterns between sapwood and heartwood in *Eucalyptus urophylla*, confirming the method’s sensitivity to chemical composition of different regions of the wood. Near-infrared (NIR) spectroscopy proved effective in identifying key wood constituents, offering a rapid, direct, and environmentally friendly alternative to conventional chemical analyses. The results support FieldSpec as a robust tool for chemical evaluation, enabling significant reductions in analysis time and eliminating the need for hazardous reagents. Continuous improvement of this technique can increase efficiency, safety, and quality control in the wood preservation industry and other industrial sectors, such as biofuels, healthcare, food, that use cellulose and lignin as biopolymers in their applications.

## Figures and Tables

**Table 1:** Descriptive statistics of chemical components (lignin and cellulose) across sections, species (SEU, SP, and HEU) and pressures (*n* = 180).

	Parameter	Count (*n*)	Mean	*SD* (%)	Median	Minimum	Maximum	*CV* (%)
**TOP**	Lignin (%)	180	30.1	8.4	29.7	9.7	57.9	28.1
	Cellulose (%)	180	2.4	1.5	2.3	0.4	7.2	61.2
**MIDDLE**	Lignin (%)	180	29.6	8.9	29.2	12.4	57.7	30.3
	Cellulose (%)	180	2.4	1.4	2.4	0.2	6.5	59.5
**BASE**	Lignin (%)	180	27.7	8.1	26.7	13.5	57.1	29.1
	Cellulose (%)	180	2.3	1.3	2.0	0.1	6.7	58.5

*n*: number of samples; *SD*(%): standard deviation; *CV*(%): coefficient of variation.

**Table 2:** *PLS* regression models, calibration, and cross-validation statistics for the cellular components (lignin and cellulose) measured from different wood species (SEU, SP, and HEU). The table includes the maximum *PLS* factor, coefficients of determination (*R*^2^), bias, root mean square error (*RMSE*), and ratio between prediction and bias (*RPD*) for each parameter during both calibration and cross-validation (*n* = 180).

Sensors	Parameter	Maximum Factor *PLS*	Calibration				Cross-Validation			
			*R* ^2^	Offset	*RMSE*	*RPD*	*R* ^2^	Offset	*RMSE*	*RPD*
TOP	**Lignin (mg/g)**	4	0.96	1.4	0.1	3.61	0.95	2.5	0.2	3.17
	**Cellulose (nmol/mg)**	6	0.99	0.0	0.3	6.01	0.98	0.1	0.3	4.93
MIDDLE	**Lignin (mg/g)**	5	0.96	1.6	0.1	3.43	0.94	2.3	0.2	3.01
	**Cellulose (nmol/mg)**	7	0.99	0.0	0.2	8.02	0.99	0.1	0.2	6.30
BASE	**Lignin (mg/g)**	5	0.96	1.3	0.1	3.74	0.94	2.8	0.1	2.94
	**Cellulose (nmol/mg)**	7	0.99	0.0	0.2	7.77	0.98	0.1	0.3	4.80

**Table 3:** Predictive statistical parameters were obtained from *PLS* regression models for the cellular components (lignin and cellulose) measured in different wood species (SEU, SP, and HEU). The table presents the maximum *PLS* factor, correlation coefficient (*r*), coefficient of determination (*R*^2^), slope, the deviation, root mean square error (*RMSE*), ratio between the prediction and deviation (*RPD*). Additionally, it includes the deviation and the linear equation that relates the predictions to the calibration model (*R*^2^*P*). The total sample size is *n* = 180.

Sensors	Parameter	Maximum Factor *PLS*	Predicted								
			*r*	*R* ^2^	Slope	Offset	*SEP*	*RMSEP*	*RPD*	Bias	Linear Equation Prediction to Calibration Model (*R*^2^*P*)
TOP	**Lignin (nm)**	4	0.94	0.92	1.02	–0.7	0.1	0.1	2.93	−0.06	Y = 0.9241X + 2.8275
	**Cellulose (nm)**	6	0.98	0.97	0.89	0.3	0.3	0.3	4.65	0.04	Y = 1.0933X − 0.2927
MIDDLE	**Lignin (nm)**	5	0.96	0.96	0.99	0.4	0.1	0.1	3.67	0.0	Y = 0.9719X + 0.9936
	**Cellulose (nm)**	7	0.99	0.99	0.98	0.1	0.2	0.2	8.35	0.0	Y = 1.0103X − 0.0515
BASE	**Lignin (nm)**	5	0.94	0.93	1.00	0.2	0.1	0.1	2.85	0.0	Y = 0.8665X + 4.8479
	**Cellulose (nm)**	7	0.99	0.99	1.00	0.0	0.1	0.1	8.18	0.0	Y = 0.9931X − 0.0018

## Data Availability

The raw data supporting the conclusions of this article will be made available by the authors on request.

## Abbreviations

The following abbreviations are used in this manuscript:

SEU	Sapwood of *Eucalyptus urophylla*
SP	Sapwood of *Pinus taeda*
HEU	Heartwood of *Eucalyptus urophylla*
*r*	Correlation coefficient
*PLS*	Partial Least Squares
*R*^2^	Coefficient of determination
*RMSE*	Root mean square error
*RPD*	Ratio between the prediction and deviation

## Appendix A

### Appendix A.1

**Table A1:** Description of wood treatments by sections (top, middle and base), pressures 0.10, 2.5, 5.0, 7.5, and 10 MPa) and portion (sapwood and heartwood *E. urophylla*) and sapwood *P. taeda*.

Samples (Top)		Samples (Middle)		Samples (Base)	
**A-Alb-C**	Top of untreated *E. urophylla* sapwood wood (control)	**M-Alb-C**	Middle of untreated *E. urophylla* sapwood wood (control)	**B-Alb-C**	Base of untreated *E. urophylla* sapwood wood (control)
**A-Alb-atm**	Top of *E. urophylla* sapwood impregnated at 0.10 MPa	**M-Alb-atm**	Middle of *E. urophylla* sapwood impregnated at 0.10 MPa	**B-Alb-atm**	Base of *E. urophylla* sapwood impregnated at 0.10 MPa
**A-Alb-25**	Top of *E. urophylla* sapwood impregnated at 2.5 MPa	**M-Alb-25**	Middle of *E. urophylla* sapwood impregnated at 2.5 MPa	**B-Alb-25**	Base of *E. urophylla* sapwood impregnated at 2.5 MPa
**A-Alb-50**	Top of *E. urophylla* sapwood impregnated at 5.0 MPa	**M-Alb-50**	Middle of *E. urophylla* sapwood impregnated at 5.0 MPa	**B-Alb-50**	Base of *E. urophylla* sapwood impregnated at 5.0 MPa
**A-Alb-75**	Top of *E. urophylla* sapwood impregnated at 7.5 MPa	**M-Alb-75**	Middle of *E. urophylla* sapwood impregnated at 7.5 MPa	**B-Alb-75**	Base of *E. urophylla* sapwood impregnated at 7.5 MPa
**A-Alb-100**	Top of *E. urophylla* sapwood impregnated at 10 MPa	**M-Alb-100**	Middle of *E. urophylla* sapwood impregnated at 10 MPa	**B-Alb-100**	Base of *E. urophylla* sapwood impregnated at 10 MPa
**A-Pin-C**	Top of untreated *P. taeda* sapwood wood (control)	**M-Pin-C**	Middle of untreated *P. taeda* sapwood wood (control)	**B-Pin-C**	Base of untreated *P. taeda* sapwood wood (control)
**A-Pin-atm**	Top of *P. taeda* sapwood impregnated at 0.10 MPa	**M-Pin-atm**	Middle of *P. taeda* sapwood impregnated at 0.10 MPa	**B-Pin-atm**	Base of *P. taeda* sapwood impregnated at 0.10 MPa
**A-Pin-25**	Top of *P. taeda* sapwood impregnated at 2.5 MPa	**M-Pin-25**	Middle of *P. taeda* sapwood impregnated at 2.5 MPa	**B-Pin-25**	Base of *P. taeda* sapwood impregnated at 2.5 MPa
**A-Pin-50**	Top of *P. taeda* sapwood impregnated at 5.0 MPa	**M-Pin-50**	Middle of *P. taeda* sapwood impregnated at 5.0 MPa	**B-Pin-50**	Base of *P. taeda* sapwood impregnated at 5.0 MPa
**A-Pin-75**	Top of *P. taeda* sapwood impregnated at 7.5 MPa	**M-Pin-75**	Middle of *P. taeda* sapwood impregnated at 7.5 MPa	**B-Pin-75**	Base of *P. taeda* sapwood impregnated at 7.5 MPa
**A-Pin-100**	Top of *P. taeda* sapwood impregnated at 10 MPa	**M-Pin-100**	Middle of *P. taeda* sapwood impregnated at 10 MPa	**B-Pin-100**	Base of *P. taeda* sapwood impregnated at 10 MPa
**A-Cer-C**	Top of untreated *P. taeda* sapwood wood (control)	**M-Cer-C**	Middle of untreated *P. taeda* sapwood wood (control)	**B-Cer-C**	Base of untreated *P. taeda* sapwood wood (control)
**A-Cer-atm**	Top of *E. urophylla* heartwood impregnated at 0.10 MPa	**M-Cer-atm**	Middle of *E. urophylla* heartwood impregnated at 0.10 MPa	**B-Cer-atm**	Base of *E. urophylla* heartwood impregnated at 0.10 MPa
**A-Cer-25**	Top of *E. urophylla* heartwood impregnated at 2.5 MPa	**M-Cer-25**	Middle of *E. urophylla* heartwood impregnated at 2.5 MPa	**B-Cer-25**	Base of *E. urophylla* heartwood impregnated at 2.5 MPa
**A-Cer-50**	Top of *E. urophylla* heartwood impregnated at 5.0 MPa	**M-Cer-50**	Middle of *E. urophylla* heartwood impregnated at 5.0 MPa	**B-Cer-50**	Base of *E. urophylla* heartwood impregnated at 5.0 MPa
**A-Cer-75**	Top of *E. urophylla* heartwood impregnated at 7.5 MPa	**M-Cer-75**	Middle of *E. urophylla heartwood* impregnated at 7.5 MPa	**B-Cer-75**	Base of *E. urophylla* heartwood impregnated at 7.5 MPa
**A-Cer-100**	Top of *E. urophylla* heartwood impregnated at 10 MPa	**M-Cer-100**	Middle of *E. urophylla* heartwood impregnated at 10 MPa	**B-Cer-100**	Base of *E. urophylla* heartwood impregnated at 10 MPa
